# Improved Selectivity of CeMnOx/Pt@SnO_2_ Laminated MOS Sensor for Hydrogen Cyanide Under Temperature Dynamic Modulation

**DOI:** 10.3390/nano15030155

**Published:** 2025-01-21

**Authors:** Yadong Liu, Yelin Qi, Wen Yang, Tengbo Ma, Shunping Zhang, Ting Liang

**Affiliations:** 1 Institute of NBC Defence, Beijing 102205, China; liuyadong202412@163.com (Y.L.); fhqiyelin@163.com (Y.Q.); yangwen_bnu@163.com (W.Y.);; 2 State Key Laboratory of Material Processing and Die & Mould Technology, Department of Materials Science and Engineering, Huazhong University of Science and Technology, Wuhan 430074, China; pszhang@mail.hust.edu.cn

**Keywords:** MOS sensor, HCN, selectivity, characteristic peak

## Abstract

Poor selectivity is one of the main bottlenecks restricting the development of metal oxide semiconductor (MOS) sensors. In this paper, using hydrogen cyanide (HCN) as the target gas, CeMnOx as the catalytic layer material and Pt@SnO_2_ as the gas-sensitive layer material, we have proposed a scheme to improve the selectivity of a catalytic layer/gas-sensitive layer-laminated MOS sensor under dynamic temperature modulation. We tested HCN and 12 kinds of battlefield environment simulation gases, and the results showed that the CeMnOx/Pt@SnO_2_ sensor, under the condition of temperature dynamic modulation (a constant temperature of 400 °C for the gas-sensitive layer and a variable temperature of room temperature to 400 °C for the catalytic layer; the heating and cooling rates were 200 °C/s, the highest temperature was maintained for 2 s, and the lowest temperature was maintained for 2 s), distinct characteristic peaks appeared on the G-T curves of the resistance response to HCN only. The quantification of the characteristic peaks was performed by peak heights, and the peak height of 5 mg/m^3^ HCN was obtained up to 0.104, while the peak heights of the other gases at the same concentration were up to 0.034. The peak height of HCN was significantly higher than that of other gases, which verified the high selectivity of the sensor for HCN. Meanwhile, the sensor also showed good sensitivity, response/recovery time, stability and anti-interference for HCN under the above temperature dynamic modulation. This work provides an important reference for the selectivity improvement of MOS sensors for HCN.

## 1. Introduction

Hydrogen cyanide (HCN), as a systemic chemical agent, can be loaded into artillery shells, bombs, or aerial dispersal for release [1,2]. Humans can be mildly poisoned by inhalation of 10–20 mg/m^3^ for a few hours. From laboratory-based equipment to vehicle-mounted and then handheld equipment [3,4,5], the testing equipment for HCN is getting smaller and lighter to meet the need for portability in the actual testing environment. Metal oxide semiconductor (MOS) sensors have the advantages of high integration, small size and light weight, and can be used as portable sensors [6,7,8,9]. Therefore, the application of MOS sensors to the detection of HCN is of great research value.

Poor selectivity is one of the main bottlenecks constraining the development of MOS sensors [10], and researchers have improved the selectivity of MOS sensors through doping and modification of noble metals [11], construction of heterojunctions [12], morphology optimization [13], temperature modulation [14] and gas-sensing arrays [15]. Choi et al. [16] synthesized Al-doped TiO_2_ via the auto-ignition method, and the Al-doped TiO_2_ showed considerable selectivity and sensitivity to CO at a temperature of 600 °C compared to undoped TiO_2_. Madhukar Poloju et al. [17] prepared pure ZnO nanoparticles and Al-ZnO/CuO heterostructured nanomaterials by the sol–gel method and the results showed that the Al-ZnO/CuO sensor had an excellent resistance response to 500 × 10^−6^ NH_3_ at room temperature compared to the pure ZnO sensor, as well as good stability, a fast response time (14 s) and a fast recovery time (9 s). Hassan et al. [18] prepared a 3D graphene aerogel hybrid material modified with macroporous ZnO nanoparticles (NPs), attributed to the strong interface between graphene and ZnO and the increase in the number of active sites available on the surface of graphene, which showed good selectivity for NO_2_ at low concentrations. Wang et al. [14] determined that the Ag/ZnO sensor exhibits excellent selectivity for CO and CH_4_ at 130 °C and 200 °C, respectively, through continuous experimentation and optimization of the heating temperature of the sensor. Duan et al. [19] devised a sensor array encompassing four distinct W-CeO_2_ sensors and the discrimination accuracy for qualitative (100%) and quantitative (89%) classification of H_2_S, ethanol, and their mixtures within a high humidity range. However, there are relatively few reports on the selectivity improvement of HCN by MOS sensors. The combination of laminated structures and temperature dynamic modulation is currently an effective approach to improve the selectivity of MOS sensors [20]. This improvement allows the MOS sensor to produce characteristics in the resistance response curves of the target gas that distinguish it from interfering gases. This approach is more suitable for gases such as HCN that require accurate identification than most of the evaluation of high selectivity by high resistance response.

Temperature dynamic modulation is an effective way to solve the first problem [21]. Temperature dynamic modulation can magnify the difference between the signals produced by different gases at different temperatures, making it easier to distinguish and identify the signals of the target gas. For example, for multiple gases with similar static responses, temperature modulation makes them have different peaks, valleys, or trends during the dynamic response to improve signal discrimination and thus improve the selectivity of the sensor for the target gas. Parret et al. [22] have investigated the effect of short-time temperature changes on the transient response of a nano SnO_2_ sensor. Even at high concentrations of C_3_H_8_ and NO_2_, different concentrations of CO can be successfully distinguished by normalizing the shape and time constant of the response curve.

The construction of a catalytic layer/gas-sensitive layer-laminated structure is an effective way to solve the second problem [23]. In a catalytic layer/gas-sensitive layer MOS sensor, the catalytic layer can be used to pretreat the gas to increase the sensitivity to the target gas or to reduce interference from other gases. Jeong et al. [24] prepared a CeO_2_/SnO_2_ sensor using CeO_2_ as the catalytic layer and SnO_2_ as the gas-sensitive layer, which was attributed to the ability of the CeO_2_ catalytic layer to catalytically oxidize high-activity interfering gases to low-activity or inactive forms, and the sensor showed high selectivity for volatile aromatic hydrocarbons.

**Figure 1:**
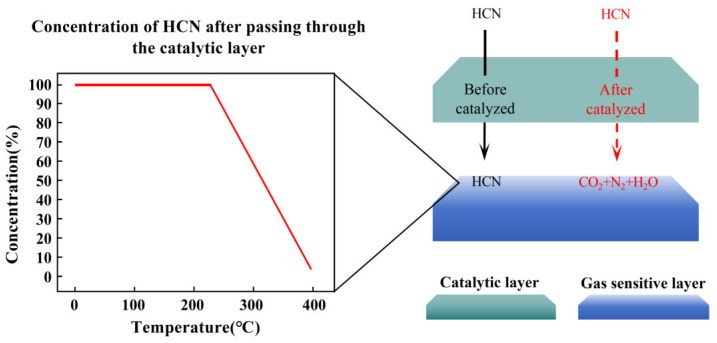
Gas concentration simulation diagram.

In this paper, a scheme combining a catalytic layer/gas-sensitive layer MOS sensor with temperature dynamic modulation was proposed to improve the selectivity of the MOS sensor. Taking HCN as the target gas, the gas-sensitive layer is at a constant temperature of 400 °C, and the catalytic layer is heated from room temperature to 400 °C; see Figure 1. The catalytic efficiency of the catalytic layer for HCN is affected by temperature and there exists an catalytic temperature. In the interval from room temperature to catalytic temperature, the catalytic ability of the catalytic layer of HCN is very weak, so HCN will not be decomposed after passing through the catalytic layer, and almost all of it will reach the surface of the gas-sensitive layer. In the interval from the catalytic temperature to 400 °C, the catalytic activity of the catalytic layer to HCN gradually increases, and HCN is decomposed after passing through the catalytic layer, resulting in a gradual decrease in the concentration of HCN when it reaches the surface of the gas-sensitive layer. It is worth noting that the increase in the temperature of the catalytic layer will increase the temperature of HCN after passing through the catalytic layer, resulting in an enhancement in the thermal motion of HCN and increasing the probability of contact with gas-sensitive layer. However, compared with the rapid response of the gas-sensitive layer to HCN at 400 °C, the change in the concentration of HCN on the surface of the gas-sensitive layer due to the enhancement of its thermal motion is negligible. The gas-sensitive layer can detect the HCN after passing through the catalytic layer and convert the catalytic information generated by the catalytic layer on HCN into a resistance signal. Since the catalytic layer produces different catalytic reactions for different gases, it is expected to achieve high selectivity of the sensor for HCN by obtaining the unique catalytic information of the catalytic layer for HCN.

According to the above scheme of selectivity improvement, a targeted selection of catalytic and gas-sensitive layer materials is required. The gas-sensitive layer material is required to have good sensitivity to HCN, due to the fact that a key factor for the peak signal to be able to occur is that the gas-sensitive layer can respond quickly and accurately to changes in the concentration of HCN on its surface. Due to the better gas adsorption ability and higher electron mobility of SnO_2_ as n-type semiconductor, the semiconductor devices based on SnO_2_ have a faster response to gases and higher electrical conductivity. The response of SnO_2_ to reducing gases has undergone more extensive study. When reducing gases are adsorbed onto the surface of SnO_2_, they will undergo a redox reaction with SnO_2_, which increases the carrier concentration of tin dioxide, leading to a decrease in its resistance value [25]. SnO_2_ is one of the most widely used MOS materials for the detection of HCN, and several experimental results have shown that SnO_2_ can effectively improve the gas sensitive response to HCN and its simulants through noble metal doping or modification [26]. Choi et al. [27] modified SnO_2_ with Pt as the gas sensitive material and further prepared a thin layer MOS sensor, which showed good sensitivity to HCN simulant at 300 °C. The catalytic layer material is required to have good catalytic ability for HCN and the gas-sensitive layer should be as insensitive as possible to the catalytic products, which is another key factor that is required the peak signals to be able to appear. Transition metal oxide catalysts are commonly used as catalysts for the conversion of hazardous gas [28,29]. Neatu S. et al. [30] prepared a CeO_2_-MnOx catalyst with excellent performance and demonstrated that the catalyst can produce catalytic combustion of CH_4_ at temperatures above 200 °C. Wang et al. [31] obtained similar conclusions that, under certain concentrations of H_2_O and O_2_, cerium oxide and manganese oxide can catalytically oxidize HCN to CO_2_, N_2_ and H_2_O.

Compared with our previous research work [20], this paper applied the catalytic layer/gas-sensitive layer-laminated MOS sensor combined with temperature dynamic modulation for the detection of a chemical agent, and further clarified the design idea of selective improvement according to this this approach. Furthermore, a new type of catalytic layer material was selected according to the properties of HCN. CeMnOx/Pt@SnO_2_ laminated structure MOS sensors were prepared by using Pt-modified SnO_2_ (Pt@SnO_2_), which is highly sensitive to HCN, as gas-sensitive layer material, and cerium oxide and manganese oxide (CeMnOx), which is of good catalytic ability for HCN, as a catalytic layer material. The special peak signals generated by the MOS sensor for HCN under certain conditions of temperature dynamic modulation have been utilized to achieve the improvement of selectivity for HCN.

## 2. Materials and Methods

### 2.1. Preparation of Layer Materials

#### 2.1.1. Preparation of Pt@SnO_2_ Slurry

The precursor was obtained by dispersing 6 mmol SnCl_4_·5H_2_O in 50 benzyl alcohol and stirring for 1 h. The reaction was carried out in a 250 mL autoclave for 24 h at 160 °C. After drying, the precursor was washed twice with a mixture of anhydrous ethanol and dichloromethane, and then washed twice with purified water, and then burned in an oven at 450 °C for 2 h to obtain SnO_2_ matrix powder. Next, 2 g SnO_2_ matrix powder was dispersed in 20 mL anhydrous ethanol, and 0.5 wt% 0.01 g/mL H_2_PtCl_6_·6H_2_O ionic solution was added according to the ratio of Pt:SnO_2_, stirred for 30 min, and then dried in an oven at 60 °C under ambient conditions. The 0.5 wt% Pt^4+^-modified SnO_2_ powder was obtained according to the above steps. The Pt@SnO_2_ slurry was obtained by adding 3 g Pt@SnO_2_ powder to 9 mL organic slurry and ball milling at 400 rpm for 4 h.

#### 2.1.2. Preparation of CeMnOx Slurry

In the next stage, 31.25. mmol Ce(NO_3_)_3_·6H_2_O and 31.25 mmol MnCI_2_·3H_2_O were dissolved in 50 mL water at 25 °C, then 0.031 g urea and 3.125 mmol polyvinylpyrrolidone k-30 (PVP) were added. Afterwards, 3.125 mL NH_3_·H_2_O was added to the above solution and stirred vigorously for 40 min, then placed in a 250 mL autoclave at 100 °C for 24 h. The resulting solid sample was immersed in NaOCI solution (5000 ppm) for 2 h then washed with anhydrous ethanol and distilled water 3 times, dried, and then calcined at 500 °C for 2 h to obtain CeMnOx powder. After that, 10 wt% CeMnOx powder was added to alumina dispersion according to CeMnOx:Al_2_O_3_ and ball milled at 400 rpm for 4 h. After drying, it was ground into powder to obtain CeMnOx loaded with Al_2_O_3_ powder. CeMnOx slurry was obtained by adding 3 g CeMnOx loaded with Al_2_O_3_ powder to 9 mL organic slurry and ball milling at 400 rpm for 4 h.

### 2.2. Preparation of the Catalytic Layer/Gas-Sensitive Layer MOS Sensor

**Figure 2:**
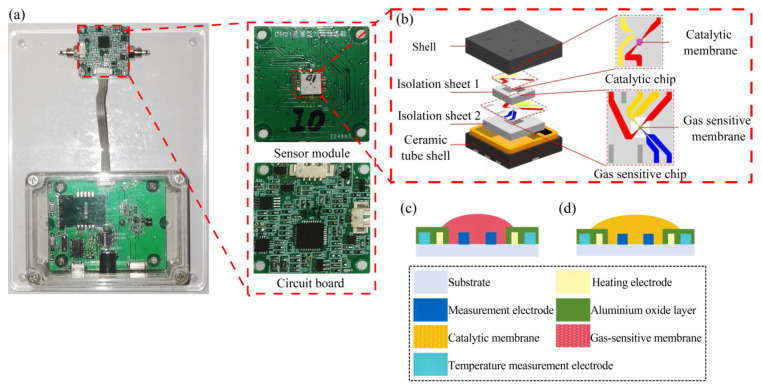
(**a**) A physical diagram of the MOS sensor; (**b**) the sensing module; (**c**) the design of the gas-sensitive chip; and (**d**) the design of the catalytic chip.

Figure 2a shows the physical diagram of the MOS sensor used in the experiment, and the sensing module is shown in Figure 2b. Figure 2c shows the design of the gas-sensitive chip and Figure 2d shows the design of the catalytic chip, the substrate is zirconium oxide with good mechanical strength. Since the MOS material is affected by the working temperature, we have chosen Pt as the heating electrode with good thermal stability and good linearity between temperature and resistance. In order to obtain the resistance change in MOS material more quickly and accurately, we chose Au with lower resistivity as the measurement electrode.

First, an electrofluidic microspray process was utilized to spray Pt@SnO_2_ slurry at the measurement electrode site of the gas-sensitive layer chip and CeMnOx slurry at the measurement electrode site of the catalytic layer chip using a 100 μm aperture needle. Then, the sprayed chips were fired in a muffle furnace at 350 °C and 550 °C for 2 h each. Finally, the fired chips were soldered and packaged to make MOS sensors that could be used for testing.

### 2.3. Test Platform

**Figure 3:**
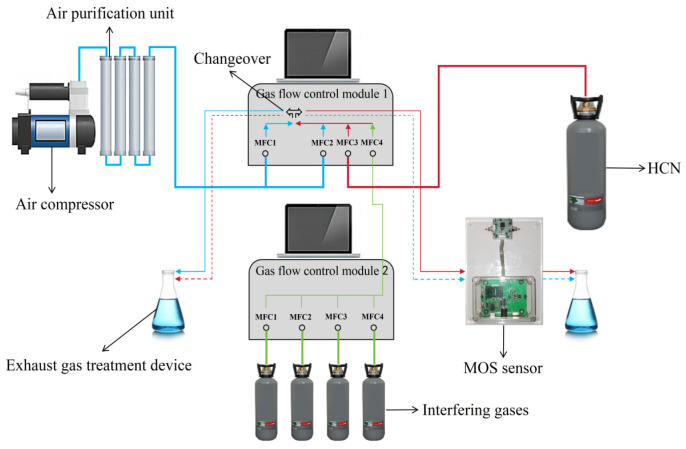
Test platform.

The test platform is shown in Figure 3. Air is supplied by an air compressor, and through the air purification device, the purified air is divided into two paths, MFC1 and MFC2 (referred to as 1-MFC1 and 1-MFC2) in gas flow control module 1. Hydrogen cyanide is supplied by gas cylinders, and enters MFC3 (referred to as 1-MFC3) in the gas flow control module 1. The interfering gases are supplied by gas cylinders, and the interfering gases enter MFC1, MFC2, MFC3, and MFC4 (referred to as 2-MFC1, 2-MFC2, 2-MFC3, and 2-MFC4) in the gas flow control module 2. Each of the above gases can be adjusted for flow size. The interfering gases are mixed after passing through 2-MFC1, 2-MFC2, 2-MFC3, and 2-MFC4, and the mixed interfering gases enter MFC4 (referred to as 1-MFC4) in gas flow control module 1. After mixing, 1-MFC2+1-MFC3+1-MFC4 will form a toxic agent path and 1-MFC1 will form an air path. Switching the toxic agent path with the air path can be realized by controlling the changeover, which alternately enters the MOS sensor through a pipe of approximately 0.3 m and the exhaust gas treatment device, as shown in the dashed lines of the figure.

Considering that humidity has a large effect on MOS sensors, it is not conducive to accurate testing of performance. Therefore, sufficient desiccant is added to the air purification unit in the test platform. In addition, the test units of the MOS sensors are all in closed pipelines to avoid the influence of water and other interfering gases in the air on the performance of the MOS sensors.

HCN was purchased from Clean Energy Technology Co., Ltd. (Fushun, China) at a concentration of 25 ppm with nitrogen as the carrier gas. Interference gases were purchased from Clean Energy Technologies Ltd. at a concentration of 80 ppm, and the carrier gas was nitrogen.

### 2.4. Battlefield Environment Simulation Gases

Based on the results of a study published in 1999 by the Chemical Division of the Naval Research Laboratory (NRL) on the composition of gases in the battlefield environment [32], the main gases present are toluene, water, diesel fuel exhaust, aviation fuel exhaust, ethylene dichloride, vegetation-burning smoke, bleach vapors, ammonia, sulfur dioxide, and isopropanol. Through further review of the data, the main components of diesel and aviation fuel exhaust, vegetation-burning smoke, and bleach vapors are shown in Table 1. Since the air itself contains a certain amount of carbon dioxide, nitrogen, and water, 12 interfering gases are used in this paper to simulate the gases in a battlefield environment: carbon monoxide, nitric oxide, nitrogen dioxide, methane, ethylene, sulfur dioxide, toluene, ethylene dichloride, ammonia, hydrogen chloride, chlorine, and isopropanol.

## 3. Results and Discussion

### 3.1. Characterization of Material

**Figure 4:**
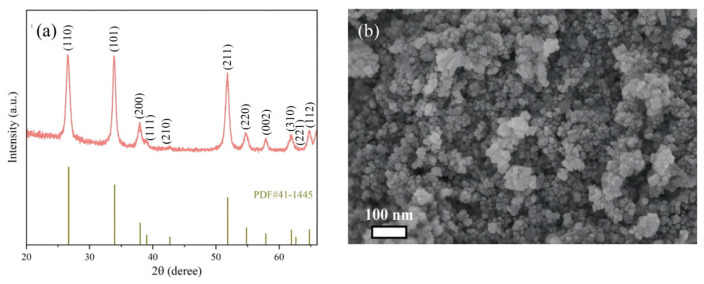
(**a**) XRD pattern and (**b**) SEM image of Pt@SnO_2_ powder.

Nano Pt@SnO_2_ powders covered with clusters of about 20 nm in diameter forming uniformly interconnected nanocrystals were synthesized by a non-aqueous sol–gel method. Figure 4a shows the XRD pattern of Pt@SnO_2_ powder, which was measured with Cu Kα radiation on a Philips X’Pert diffractometer (2θ from 10° to 80°, λ = 1.5406 Å, Amsterdam, The Netherlands). It can be seen that Pt@SnO_2_ has a high purity and crystallinity, with almost no impurity peaks. SnO_2_ can be indexed with reference to the corresponding standard card (20-1324), and because of the small amount of Pt^4+^, this figure does not clearly show the information related to these modifying elements. The SEM image of Pt@SnO_2_ powder is shown in Figure 4b.

### 3.2. Selectivity

#### 3.2.1. Appearance of Characteristic Peaks

**Figure 5:**
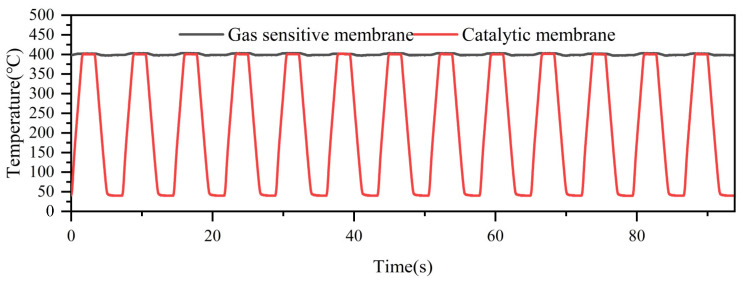
Temperature dynamic modulation.

Figure 5 demonstrates the temperature curve of the CeMnOx/Pt@SnO_2_ sensor under the temperature dynamic modulation (a constant temperature of 400 °C for the gas-sensitive layer and a variable temperature from room temperature to 400 °C for the catalytic layer; the heating and cooling rates were 200 °C/s; the highest temperature was maintained for 2 s; and the lowest temperature was maintained for 2 s). From the figure, it can be seen that the noise of the gas-sensitive layer was ±4 °C at 400 °C, the noise of the catalytic layer was ±2 °C at 400 °C, and the noise was ±2 °C at room temperature (the catalytic layer is about 40 °C at room temperature, which is due to the heating that causes the temperature inside the chamber to increase and cannot be quickly recovered to the actual room temperature), and the period of a single temperature change was about 7.2 s. It is shown that the temperature dynamic modulation of the sensor was accurate, stable, and fast.

**Figure 6:**
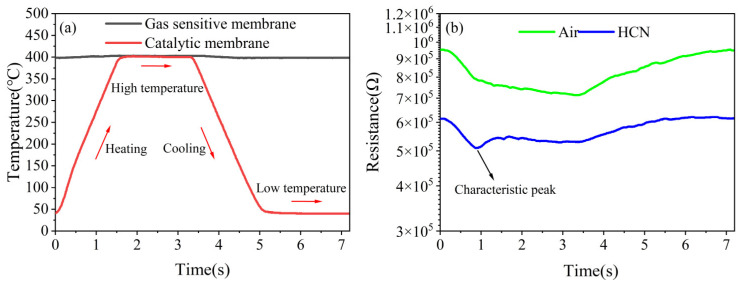
(**a**) Temperature curve and (**b**) resistance curve of the sensor in one cycle.

Figure 6a shows the temperature–time (T-t) curve of the sensor over one cycle with the above temperature dynamic modulation, and Figure 6b shows the resistance–time (R-t) curve of the sensor over one cycle for air and 5 mg/m^3^ HCN. When the sensor was in an air atmosphere, it could be observed that when the catalytic layer was heating, the resistance decreased; when the catalytic layer was kept at the high temperature, the resistance continued to decrease slowly to the lowest amount; when the catalytic layer was cooling, the resistance increased; and when the catalytic layer was kept at the low temperature, the resistance continued to increase to the highest amount. According to the adsorption/desorption model [33], when the temperature of the catalytic layer increased, the temperature of the oxygen passing through the catalytic layer increased, and the activity was enhanced, increasing the adsorption on the surface of the gas-sensitive layer and seized the electrons of the gas-sensitive layer, causing the resistance of the gas-sensitive layer to decrease. When the sensor was in an HCN atmosphere, it was observed that the resistance of the sensor was decreased compared to the air atmosphere. This is due to the fact that Pt@SnO_2_ acts as an N-type semiconductor and HCN acts as a reducing gas, and the resistance of the N-type semiconductor tends to decrease when it comes into contact with the reducing gas compared to the air atmosphere.

In addition, a sharp “characteristic peak” was observed in the HCN atmosphere, which did not occur in the air atmosphere. Its location was at the heating phase rather than at the highest temperature during the process of temperature dynamic modulation. This characteristic peak may be attributed to the catalytic effect of the catalytic layer on HCN. The appearance of the characteristic peak provided a possible reference for realizing the high selectivity of the sensor for HCN.

**Figure 7:**
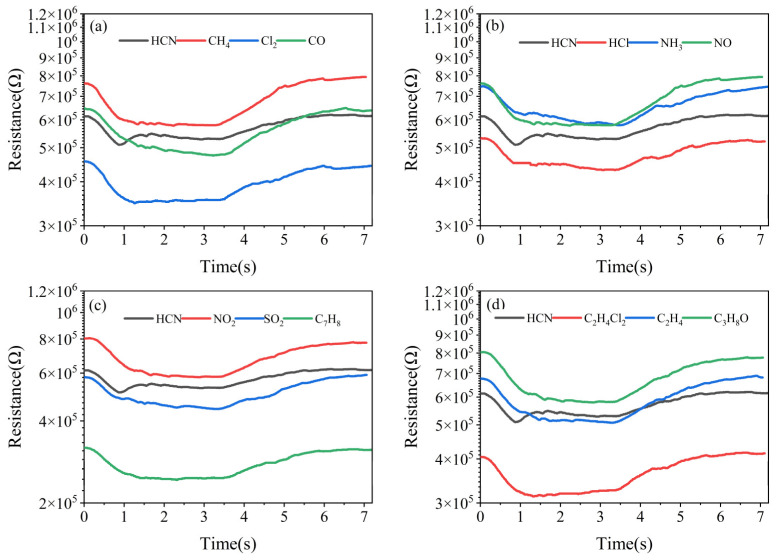
Resistance response of the sensor to (**a**) HCN, CH_4_, Cl_2_, CO; (**b**) HCN, HCl, NH_3_, NO; (**c**) HCN, NO_2_, SO_2_, C_7_H_8_; (**d**) HCN, C_2_H_4_Cl_2_, C_2_H_4_, C_3_H_8_O.

In order to verify whether the characteristic peak was the specific response of the sensor to HCN, the sensor was tested in the same way using battlefield environment simulation gases at a concentration of 5 mg/m^3^. The results, as shown in Figure 7, showed that the sensor had resistance responses to all gases, but the characteristic peak appeared only in the resistance response curve of HCN and did not appear in the resistance response curves of the other gases.

#### 3.2.2. Definition of Peak Height

**Figure 8:**
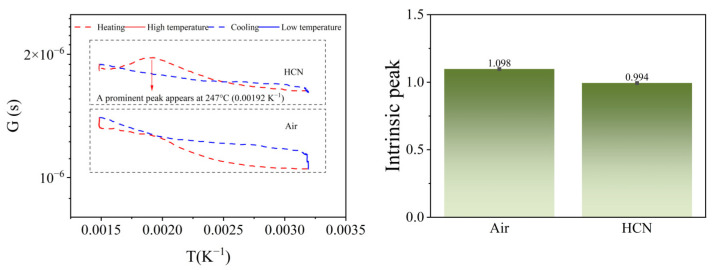
(**a**) G-T curves and (**b**) intrinsic peak heights of air, 5 mg/m^3^ HCN.

Distinguishing from most MOS sensors that quantify selectivity using sensitivity, we proposed a computational method to quantify selectivity based on the characteristic peak. By combining the T-t and R-t data into conductivity–temperature (G-T) data, the magnitude of the characteristic peak could be reasonably calculated. Figure 8 shows the G-T curves of air and 5 mg/m^3^ HCN. From Figure 8a, it can be seen that compared with the similar G-T curve of air, the G-T curve of HCN had a prominent peak at 247 °C (0.00192 K^−1^) during the heating phase, and therefore the height of the peak could be used to respond to the magnitude of the characteristic peak.

If the optimal catalytic temperature of the catalytic layer for HCN corresponded to the temperature Tc, which was the highest point of the peak of the G-T curve, the ratio of the conductance at the highest point of the peak of the G-T curve at this point in the HCN atmosphere to the bottom of the peak at the corresponding temperature could be calculated as

(1) $$S_{\mathrm{Gas}}=\frac{R_{\left(\mathrm{Gas},C,\mathrm{Tc}\right)}^{-1}}{R_{\left(\mathrm{Gas},H,\mathrm{Tc}\right)}^{-1}}=\frac{R_{\left(\mathrm{Gas},H,\mathrm{Tc}\right)}}{R_{\left(\mathrm{Gas},C,\mathrm{Tc}\right)}}$$

where R_(Gas,C,Tc)_, R^−1^_(Gas,C,Tc)_ denoted the resistance and the reciprocal of this resistance during the cooling phase to Tc in HCN atmosphere, respectively; R_(Gas,H,Tc)_, R^−1^_(Gas,H,Tc)_ denoted the resistance and the reciprocal of the resistance of the sensor during the heating phase to Tc; and S_Gas_ denotes the intrinsic peak height of HCN. Similarly, the intrinsic peak height of air at Tc was

(2) $$S_{\mathrm{Air}}=\frac{R_{\left(\mathrm{Air},C,\mathrm{Tc}\right)}^{-1}}{R_{\left(\mathrm{Air},H,\mathrm{Tc}\right)}^{-1}}=\frac{R_{\left(\mathrm{Air},H,\mathrm{Tc}\right)}}{R_{\left(\mathrm{Air},C,\mathrm{Tc}\right)}}$$

The meaning of each parameter was similar to that in HCN atmosphere, R_(Air,C,Tc)_, R^−1^_(Air,C,Tc)_ denoted the resistance and the reciprocal of this resistance when cooling to Tc in air, and R_(Air,H,Tc)_, R^−1^_(Air,H,Tc)_ denoted the resistance of the sensor and the reciprocal of the corresponding resistance when heating to Tc, respectively. Therefore, the G-T peak height of HCN was

(3) $$S_{\mathrm{Peak}}=S_{\mathrm{Gas}}-S_{\mathrm{Air}}$$

Following the defined method, the intrinsic peak height was calculated to be 0.994 of air, 1.098 of 5 mg/m^3^ HCN, and the characteristic peak height of 5 mg/m^3^ HCN was 0.104, as shown in Figure 8b.

#### 3.2.3. Selectivity Evaluation

**Figure 9:**
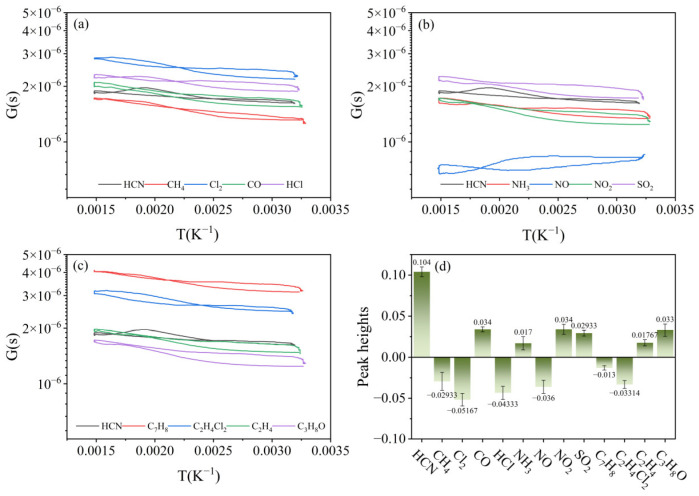
(**a**–**c**) G-T curves and (**d**) peak heights of all gases.

Figure 9a–c show the G-T curves of HCN at 5 mg/m^3^ and 12 interfering gases at the same concentration. It can be seen that when the resistance response of an interfering gas is lower than that of HCN, its G-T curve tends to shift upward compared to HCN and vice versa. We have not been able to directly compare the magnitude of the peak heights by the G-T curves in the figure, and further calculations of the peak heights of all the gases are needed.

Compared to the interfering gases, the G-T curve of HCN would have a more prominent peak at T = 0.00192 K^−1^. By calculating the peak height, the maximum value of the characteristic peak height of interfering gases was 0.034, which was obviously lower than that of the characteristic peak height of HCN (0.104), as shown in Figure 9d. This indicated that it was feasible to evaluate the high selectivity of the sensor for HCN by the characteristic peak and to use the peak height to realize the quantification of the selectivity.

We consider that compared with most sensors that use the resistance response value to evaluate the selectivity to the target gas, using the peak height of the characteristic peak to evaluate the selectivity has certain advantages. The resistance response value is easily affected by the complexity of the gas in the detection environment, the operating time of the sensor, humidity, and other factors, while the characteristic peak is a kind of overall trend of the resistance response curve, which is more capable of maintaining the original traits under the existence of the influencing factors. At the same time, the characteristic peak represents the sensor’s specific response to the target gas, and this is more suitable for HCN and other gases that need to be identified accurately.

### 3.3. Sensitivity

**Figure 10:**
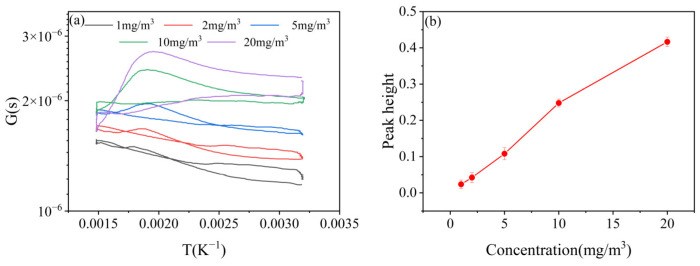
(**a**) G-T curves and (**b**) peak heights of HCN at different concentrations.

Figure 10a shows the G-T curves of the sensor for different HCN concentrations under the above temperature dynamic modulation, and they are gradually shifted upward with the increase in the HCN concentration. As shown in Figure 10b, the peak height of HCN increased gradually with the increase in the HCN concentration. When the HCN concentration was 2 mg/m^3^, the peak height was about 0.044, which was higher than the peak height of any interfering gas at 5 mg/m^3^, indicating that the sensor had better sensitivity for HCN; when the HCN concentration was as low as 0.1 mg/m^3^, the peak height was close to 0, which indicated that the sensor’s minimum detection limit for HCN was about 0.1 mg/m^3^.

### 3.4. Response/Recovery Time

**Figure 11:**
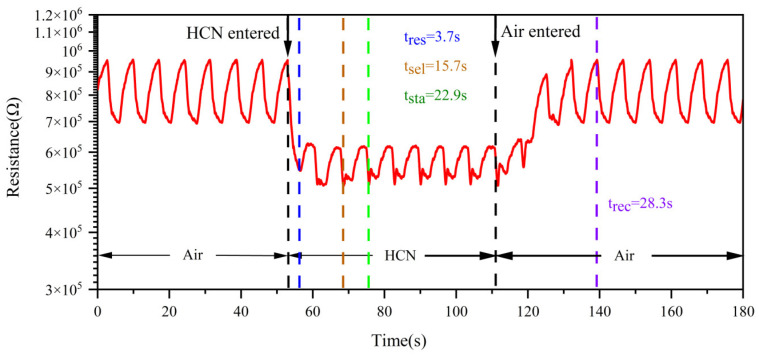
Response/recovery time of 5 mg/m^3^ HCN.

Figure 11 shows the resistance response curve of the sensor to 5 mg/m^3^ HCN under the above temperature dynamic modulation. As shown in the figure, the air was switched to HCN at 52.7 s. When HCN entered, the resistance of the sensor began to decrease, and when the response curve began to change, it indicated that the sensor had detected HCN, at which time the response time was about 3.7 s. This indicated that the sensor had a fast response to HCN. With the increase in HCN fluence time, the characteristic peaks began to appear in the response curve, and the characteristic peaks were more obvious after 15.7 s. At this time, the sensor showed high selectivity to HCN. The peak height of the characteristic peaks gradually increased with the continuous increase in HCN fluence time, and finally reached a stable state after 22.9 s. HCN was switched to air at 111.3 s. When air entered, the resistance of the sensor began to recover gradually and recovered to the steady state of air at 139.6 s. The recovery time was about 28.3 s.

At present, the alarm time of carried toxic agent detection devices such as W-ZHMG200CWA and W-BD8-CWA-MG106AH can reach less than 3 s and recovery usually occurs within minutes. The actual response time of this MOS sensor can be further shortened due to the influence of the length of the pipe, so the sensor had a fast response/recovery time.

### 3.5. Stability

**Figure 12:**
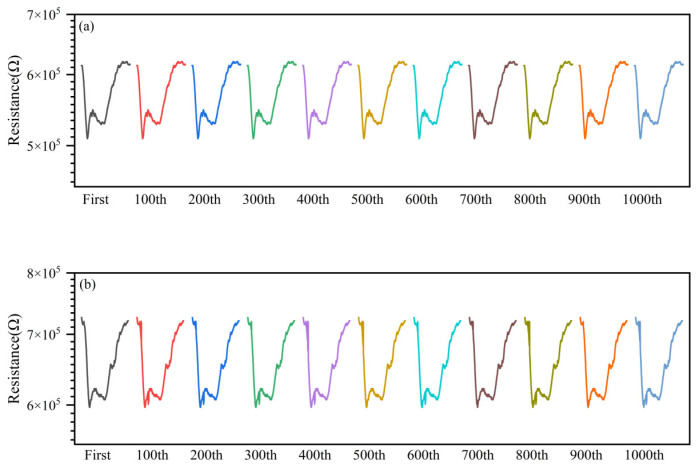
(**a**) Response of the sensor to 5 mg/m^3^ HCN and (**b**) after 133 days.

In order to test the stability of this sensor under the above temperature dynamic modulation, we continuously tested the sensor 1000 times to 5 mg/m^3^ HCN and recorded the resistance response curves (1 pulse recorded every 100 times). The test results are shown in Figure 12a. It can be seen that the resistance curve of the 1000th time was not significantly different from that of the first time, and there was almost no change to the characteristic peak of HCN. The fluctuation range of the peak height was obtained as 0.101~0.105 by calculation, which indicated that the sensor was of good stability. The same test was performed again after 133 days, and it can be seen that the characteristic peaks of the sensor for HCN have slightly decreased, but a clear characteristic peak can still be observed, and the fluctuation of the peak height was in the range of 0.085~0.093, as shown in Figure 12b.

### 3.6. Anti-Interference

The anti-interference capability of the CeMnOx/Pt@SnO_2_ sensor in complex atmospheric environments was examined. Twelve interfering gases from simulated battlefield environments were selected to assess the sensor’s anti-interference abilities against HCN. The experiments were conducted in four groups, with 5 mg/m^3^ HCN and 5 mg/m^3^ interfering gases added to each group, as shown in Table 2.

**Figure 13:**
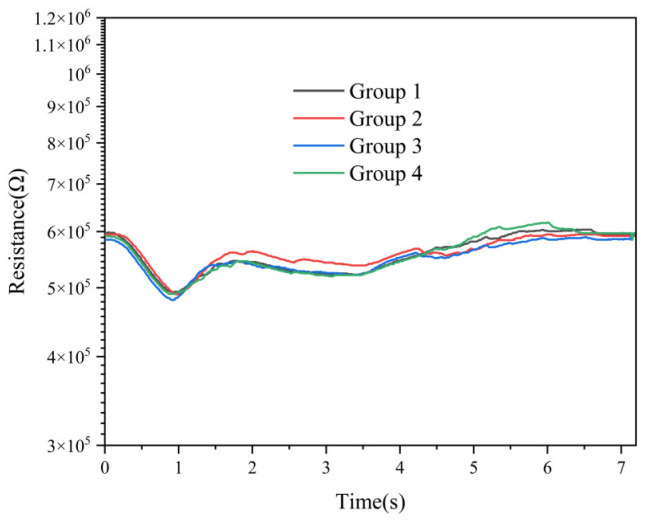
Response of the sensor to 5 mg/m^3^ HCN and 12 interfering gases.

The resistance responses of four groups of gases are shown in Figure 13. It is observed that for 12 interfering gases, the sensor still had obvious characteristic peaks for HCN, indicating that the sensor had good anti-interference ability for HCN.

### 3.7. Sensing Mechanisms of High Selectivity

#### 3.7.1. The Sensing Mechanism of Pt@SnO_2_ Gas-Sensitive Layer for HCN

It is well known that the layer surface is usually covered with chemisorbed oxygen ions, including $O_{-2}$, O^−^, and O^2−^. As the temperature increases, the reaction can be described as follows:

(4) $$O_{2}\left(\mathrm{ads}\right)+e^{-}↔O_{2}^{-}\left(\mathrm{ads}\right)$$

(5) $$O_{2}^{-}\left(\mathrm{ads}\right)+e^{-}↔{2O}^{-}\left(\mathrm{ads}\right)$$

(6) $$O^{-}\left(\mathrm{ads}\right)+e^{-}↔O^{2-}\left(\mathrm{ads}\right)$$

$O_{-2}$, O^−^, and O^2−^ are separately formed at 25~150 °C, 150~300 °C and more than 300 °C. The interaction between HCN and oxygen ions is as follows:

(7) $$O_{2}^{-}+2\mathrm{HCN}=2\mathrm{HOCN}+e^{-}$$

(8) $$O^{-}+\mathrm{HCN}=\mathrm{HOCN}+e^{-}$$

(9) $$O^{2-}+\mathrm{HCN}=\mathrm{HOCN}+{2e}^{-}$$

**Figure 14:**
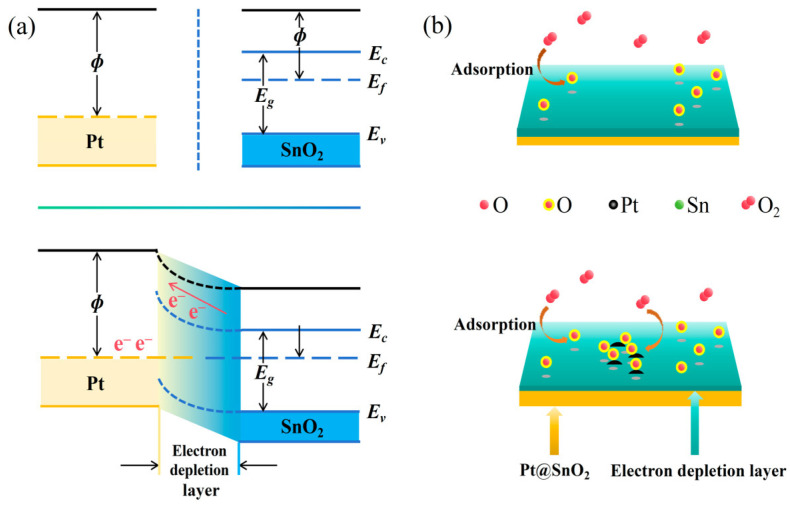
Changes in (**a**) work function and (**b**) surface activity of Pt@SnO_2_.

The reason why Pt can enhance the response of SnO_2_ to HD was analyzed based on the Schottky barrier model [34]. The work function of Pt is generally larger than that of SnO_2_, and when they come into contact, electrons will be transferred fromSnO_2_ to Pt or Rh, forming a depletion layer on the surface of SnO_2_. At this time, a Schottky barrier is created on the surface of Pt@SnO_2_ that prevents the electrons from escaping further, which increases its resistance, as shown in Figure 14a. HCN molecules adsorbed on the surface of Pt@SnO_2_ react with the oxygen on the surface to release electrons, which fill up the depletion layer on the surface of Pt@SnO_2_ and reduce the resistance of Pt@SnO_2_.

Pt nanoparticles can form new active sites on the SnO_2_ surface. On the one hand, Pt and Rh nanoparticles themselves can act as active centers and directly participate in the reaction process by adsorbing and activating HD molecules. On the other hand, they can also induce more defects and oxygen vacancies on the SnO_2_ surface, which are surrounded by atoms with higher chemical activity that can interact more strongly with reducing gases, thus increasing the reaction activity, as shown in Figure 14b.

#### 3.7.2. High Selective Catalysis of HCN by CeMnOx Catalytic Layer

**Figure 15:**
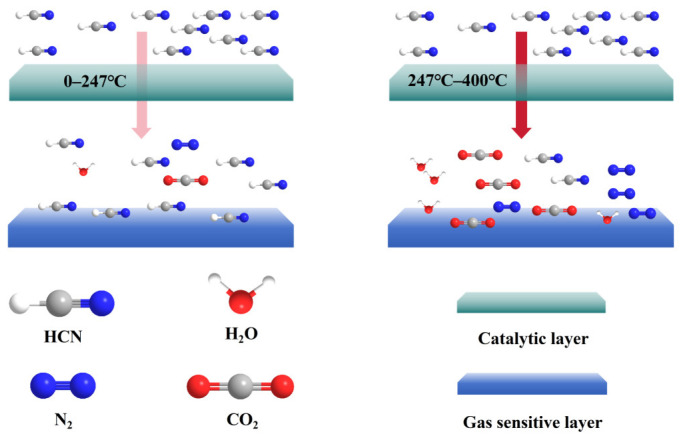
Simulation of the catalytic reaction of HCN.

Combined with the characteristic peaks of the resistances, we analyzed the catalytic function of the catalytic layer on HCN. Under the catalytic function of Pt, a redox reaction between HCN and O_2_ would occur. In the range of 0–247 °C, the catalytic ability of CeMnOx on HCN was weak, and a small amount of CO_2_+N_2_+H_2_O would be produced; when the temperature reached 247 °C, the catalytic ability of CeMnOx on HCN would be significantly enhanced, at which time the vast majority of HCN would be consumed, and a large amount of CO_2_+N_2_+H_2_O would be produced, as shown in Figure 15.

It is worth noting that not all gases can have characteristic peaks in their resistance response, but they must meet the criteria of being able to be catalyzed by the catalytic layer to a less reducing gas. For example, Simion, C. E. et al. [35] demonstrated that CH_4_ can be catalytically combusted by CeMnOx at 223 °C to produce CO_2_, H_2_O and N_2_; however, CH_4_ did not present characteristic peaks in the experiments obtained here. Therefore, we believe that another key factor for the appearance of the characteristic peaks is that the catalytic oxidation of HCN can, in most cases, be achieved quickly within a short period of time (less than 1.5 s) during the warming process by the catalytic layer.

#### 3.7.3. Generation Mechanism of the Characteristic Peak

**Figure 16:**
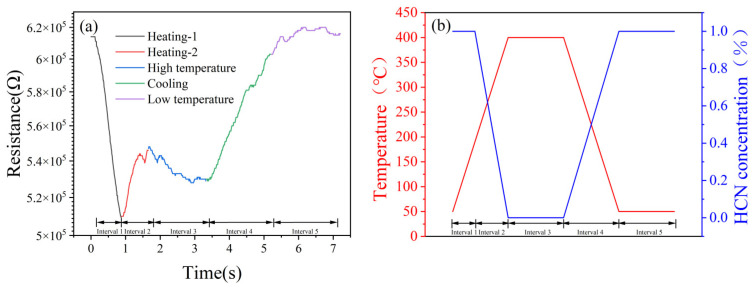
(**a**) Five intervals of a single cycle; (**b**) simulation of temperature and corresponding HCN concentration on the surface of the gas-sensitive layer during a single cycle.

Based on the catalytic layer/gas-sensitive layer-laminated structure, the generation of the characteristic peak in the response of the CeMnOx/Pt@SnO_2_ sensor to HCN was analyzed, as shown in Figure 10. A single cycle was divided into five intervals, i.e., heating-1, heating-2, high temperature, cooling and low temperature, as shown in Figure 16a, and each interval is denoted by 1–5, respectively. The simulation of the temperature of each interval in a single cycle and the corresponding HCN concentration on the surface of the gas-sensitive layer is shown in Figure 16b.

For interval 1, the catalytic activity of the CeMnOx to HCN is low during the initial temperature rise, and HCN will not be decomposed after passing through the CeMnOx. The temperature of HCN after passing through the CeMnOx increases, and the thermal movement of HCN becomes more intense. This leads to a rapid increase in the frequency of contact between HCN and the surface of the Pt@SnO_2_, and a rapid decrease in resistance. However, the rapid reaction between Pt@SnO_2_ and HCN results in no change in the HCN concentration on the Pt@SnO_2_ surface.

For interval 2, when the CeMnOx temperature increases from 247 °C to 400 °C, CeMnOx starts to have catalytic activity for HCN. Therefore, the concentration of HCN decreases after it passes through CeMnOx, which leads to the weakening of the reaction with Pt@SnO_2_, and the resistance of Pt@SnO_2_ increases, i.e., the characteristic peak appears.

For interval 3, due to the high catalytic activity of the CeMnOx to HCN at 400 °C, HCN is completely decomposed after passing through the CeMnOx. And the rapid reaction between the gas Pt@SnO_2_ and HCN results in the concentration of HCN on the surface of the Pt@SnO_2_ being almost 0. Resistance decreases slowly due to the low temperature increase, while the concentration of HCN is quasi-constant.

For interval 4, the decrease in the temperature of the CeMnOx makes it less active in catalyzing HCN, leading to an increase in the concentration of HCN when it reaches the surface of the Pt@SnO_2_ after passing through the CeMnOx. And resistance increases slowly due to the low temperature decrease.

For interval 5, CeMnOx does not have catalytic activity for HCN at room temperature, so that HCN will not be catalytically decomposed after passing through CeMnOx, resulting in the HCN concentration on the surface of Pt@SnO_2_ and the resistance being restored to maximum levels.

## 4. Conclusions

When the Pt@SnO_2_ gas-sensitive layer was heated at a constant temperature of 400 °C and the CeMnOx catalytic layer was heated from room temperature to 400 °C, the change in the catalytic activity of the CeMnOx catalytic layer for HCN resulted in a change in the state of HCN after passing through the CeMnOx catalytic layer. The Pt@SnO_2_ gas-sensitive layer can detect the changes in HCN and convert them into characteristic peaks of the resistance response. The characteristic peak was quantified by the peak height, and the peak height of HCN was significantly larger than that of 12 battlefield environment simulation gases at the same concentration, which verified that the sensor could achieve high selectivity for HCN under temperature dynamic modulation. Meanwhile, the sensor also showed good sensitivity, response/recovery time, stability, and anti-interference for HCN.

The high selectivity of the MOS sensor for HCN by the characteristic peak also has some limitations under the actual detection conditions. The first is that as the concentration of HCN decreases (especially when it is below 1 mg/m^3^), its peak height will gradually decrease and converge to other gases, resulting in an inability to recognize HCN by its characteristic peak; then, when the gases in the environment are complex, especially when there are gases similar to the catalytic reaction of HCN, it is equally likely that the characteristic peak may appear; and lastly, the catalytic layer and gas-sensitive layer materials may be affected by the increase in storage time or inadequate storage conditions, resulting in a weak resistance response to HCN, affecting the peak height of the characteristic peak or even preventing such peaks from appearing.

## Figures and Tables

**Table 1:** The main gases in the battlefield environment.

Diesel and Aviation Fuel Exhaust		Vegetation-Burning Smoke		Bleach Vapor	
Carbon Monoxide	Carbon Dioxide	Carbon Monoxide	Carbon Dioxide	Hydrogen Chloride	Chlorine
Hydrocarbon	Oxynitride	Hydrocarbon	Oxynitride		
Sulfur dioxide	Water				
Nitrogen					

**Table 2:** Gas grouping situation.

Group Number	Gas Composition			
1	HCN	Carbon monoxide	Nitric oxide	Nitrogen dioxide
2	HCN	Methane	Ethene	Toluene
3	HCN	Hydrogen chloride	Chlorine	Ammonia
4	HCN	Sulfur dioxide	Dichloroethane	Isopropyl alcohol

## Data Availability

Data are contained within the article.
